# Excavated pulmonary nodules: an unusual clinical presentation of lung metastasis in two cases

**DOI:** 10.1186/1471-2342-10-13

**Published:** 2010-06-24

**Authors:** Wafa Allam, Basma Elkhanoussi, Khaoula Alaoui, Issam Lalya, Hassan Errihani

**Affiliations:** 1 Department of Medical Oncology, National Institute of Oncology, Avenue Allal Alfassi, Hayriad, 10100 Rabat, Morrocco; 2 Pathology Department, National Institute of Oncology, Avenue Allal Alfassi, Aayriad, 10100 Rabat, Morrocco; 3 Radiation Department, National Institute of Oncology, Avenue Allal Alfassi, Hayriad, 10100 Rabat, Morrocco

## Abstract

**Background:**

Excavated pulmonary metastasis are rare. We present two cases of excavated pulmonary nodules proved to be metastases from osteosarcoma and gallblader lymphoma.

**Case presentation:**

The first one is 39-year-old man in whom cholecystectomy made the diagnosis of primary non-Hodgkin's lymphoma of the gallbladder. He presented in chest CT scan excavated nodules that had been biopsied and confirmed the diagnosis of non hodgkin lymphoma. He underwent 8 courses of chemotherapy CHOP 21 with complete remission. The second one is an 21 years old man who presented a right leg osteoblastic osteosarcoma with only excavated pulmonary nodules in extension assessment. He had 3 courses of polychemotherapy API (doxorubicin, platinum, and ifosfamide) with partial response. Unfortunately, he died following a septic shock.

Review of the literature shows that excavated pulmonary nodules as metastasis are rare but we should consider this diagnosis every time we are in front of a cancer. Chest computed tomography is the best diagnosis imaging that could make this diagnosis. Differential diagnosis between benign and malignant bullous lesions is important because surgical excision affects survival in some malignancies.

**Conclusions:**

Although pulmonary nodules are the most common cancer metastasis, a differential diagnosis of a concurrent primary malignancy should always be considered every time we have excavated lesions, even in patients with known malignant disease. Thorough chest evaluation is important, as multiple primary malignancies may occur concomitantly.

## Background

Metastatic causes of excavated pulmonary nodules are rare but must be considered in patients with a history of neoplastic disease.

Excavated metastatic pulmonary lesions are described, but in a country of epidemic tuberculosis, or in front of smokers, it is necessary to raise differential diagnosis. Sometimes, chest scann permitt to elaborate a definite diagnosis, and sometimes, we must resort to a biopsy. We report here two cases of excavated pulmonary metastasis attendant to primary malignancies.

## Case presentation

### Case 1

**Figure 1:**
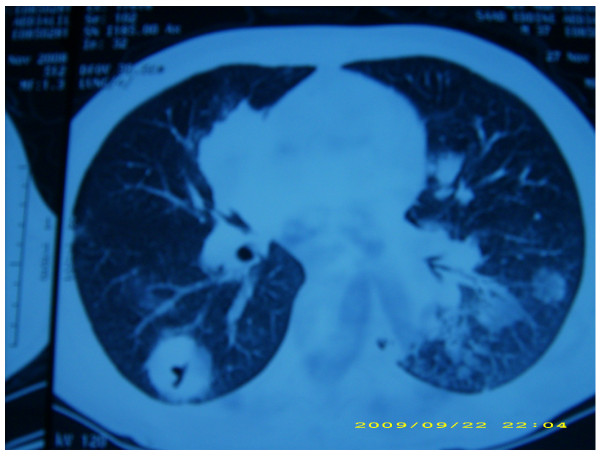
**Excavated lesions with clear limits and irregular thickened wall before chemotherapy**.

**Figure 2:**
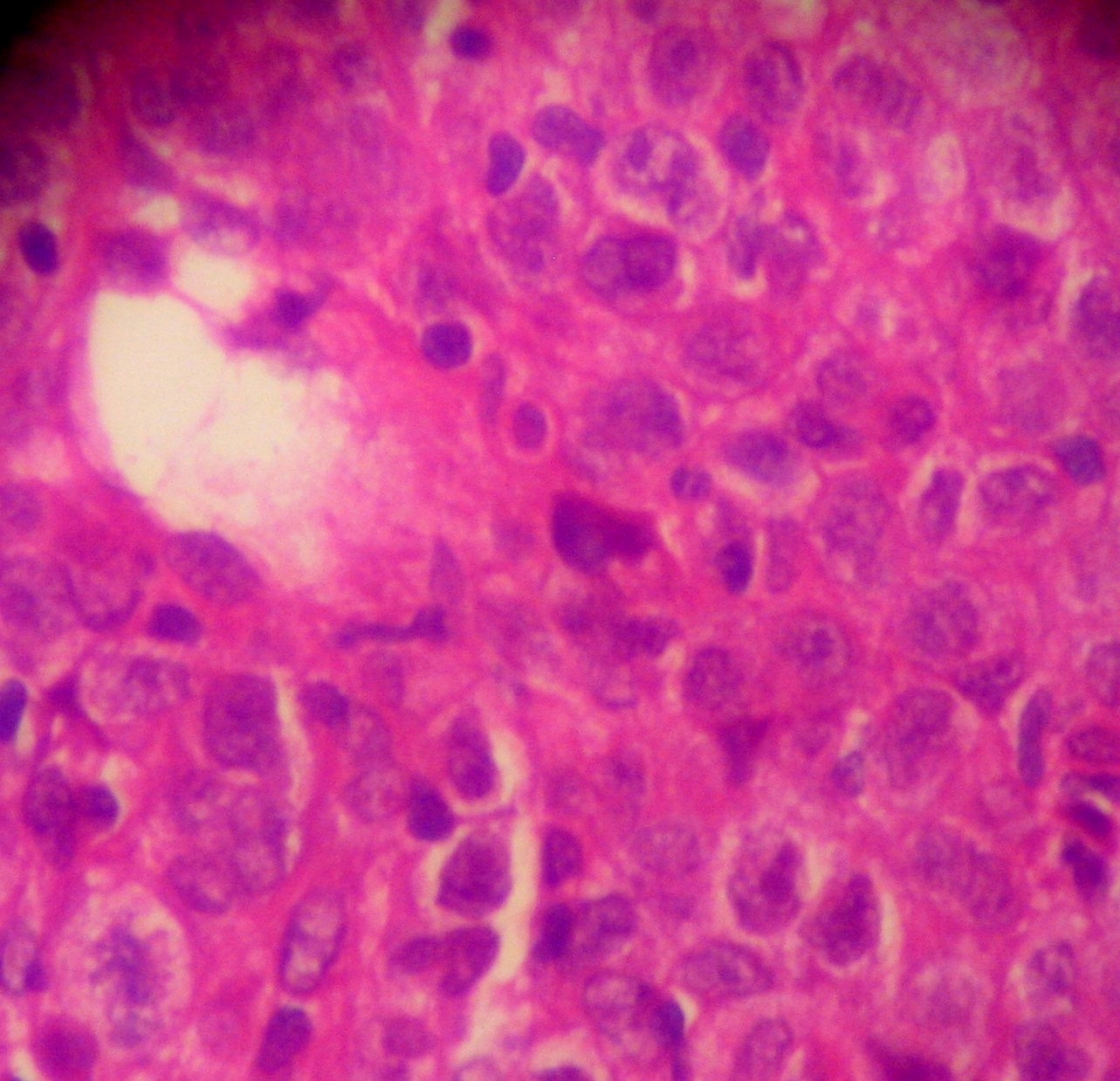
photograph with a magnification × 100 showing a large cell lymphoma proliferation

**Figure 3:**
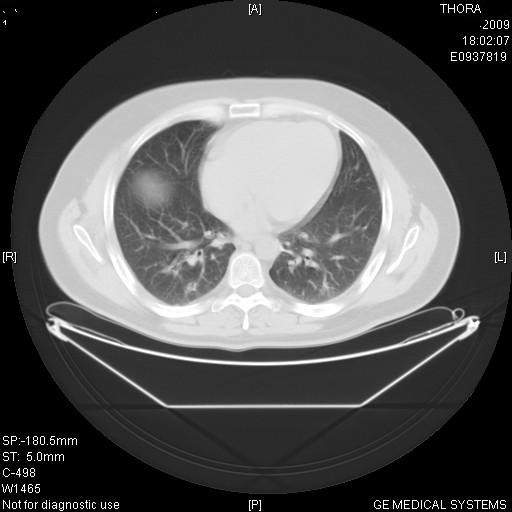
**Disappearance of lesions after chemotherapy**.

A 39 year old male with no significant past medical history, initially presented with a several month history of intermittent pain localized to the abdominal right upper quadrant. Our patient was a chronical smoker. He has consulted and a physical examination revealed multiple infracentimetric cervical lymphadenopathy. He underwent an abdominal US which was abnormal (stones within gallbaldder) and was scheduled for a cholecystectomy. Histopathology from his surgically removed gallbladder was consistent with a primary NHL T cell. Immunostaining showed a positive marking for CD45, CD3. He underwent further lymphoma staging including a computed tomography scans of the body which revealed lymph nodes with surrenal nodules and pulmonary excavated lesions (figure 1). Our patient did a bronchoscopy to confirm weither the pulmounary nodules were in relation with the NHL or a pulmnonary disease or infection such as tubeculosis and concluded to bud of the stem bronchus that was histologically a lymphoma with large cell (figure 2). All the biopsies confirmed the nature of NHL. Bone marrow bio-sy was normal. Left ventricular ejection fraction was 72%, laboratory tests were normal, except a higher rate of LDH that was 754 UI/L. According to the Ann Arbor's classification, he was staged IVBb. He underwent treatement by CHOP 21 (cyclophosphamide 750 mg/m^2 ^Intraveinously (IV) day 1, doxorubicine 50 mg IV day 1, vincristine 1,4 mg/m^2 ^IV day 1 and prednisone 40 mg/m^2 ^per day from day 1 to day 5 per os, each cycle every 21 days). He was in uncertain complete response (more than 80% according to Cheson criteria) after 4cycles and had 8 cycles of CHOP 21 with a complete response. The patient tolerated chemotherapy well and he is currently free of disease 12 months after completion of chemotherapy and 15 months from diagnosis. Response was achieved by CT scan (figure 3).

### Case 2

A 21 years old man, with no particular medical history, who presented a right leg painful mass was consulting at our hospital. There was no history of trauma or infection. On examination, a great warm mass measuring 12 × 21 cm was found at the end of the right tibia. Signs of inflammation were observed with a large swelling and oozing. He had no lymphadenopathy and no other mass. He just accused intermittent cough lasting for two months. His blood parameters were correct. An MRI (magnetic resonnance) was performed to establish the nature of this mass and its locoregional reports, it objectived abnormal signal intensities with a cortical destruction. A biopsy was then realised and the histopathological study revealed a chondroblastic osteosarcoma with an hypercellular neoplasm. He underwent an extension assessement by chest CT scan, scintigraphy, that revealed bilateral excavated pulmonary metastasis with different sizes and location. A pulmonary biopsy was performed to eliminate infectious diagnosis or vascularite, and revealed a metastasis of osteosarcoma. He had 3 courses of polychemotherapy API (doxorubicin 60 mg/m^2 ^IV day 1, cis-platinum 75 mg/m^2 ^IV day 1, and ifosfamide 7 g/m^2 ^from day 1 to 5 IV with uromitexan to protect against hemorrhagic cystitis ) with partial response. Unfortunately, he died following a septic shock.

#### Discussion

Lung represents a major metastatic site of the body as there's a seat about 30 to 50% of all secondary locations. The occurrence of excavated lung metastases is rarely observed only in 4% of cases (1). We report two cases with rare lymphoma of the gallbladder and osteosarcoma of the leg with pulmonary excavated metastasis emphasizing the role of CT in characterizing these lesions. Primitive tumor corresponds mainly to squamous cell carcinoma of the head and neck or cervix, rarely to colon (2,3). Metastatic sarcomas, especially osteogenic can disseminate as excavated pulmonary nodules with a high risk of pneumothorax. Excavation sometimes occurs after chemo-radiotherapy (4). A review of litterature shows that the primary site that metastasis to lung are excavated are bladder, kidney, breast, and ovary (2,5,6). CT scan is more sensitive than standard chest radiography for the detection of excavated metastatic lesions. It also allows aetiological diagnosis achieved by biopsy guided by the scan, as in our second observation.

## Conclusion

In conclusion, we recommend to realise chest CT scan and biopsy each suspected lesions to ensure diagnosis of metastasis.

## Consent

"Written consent was obtained from the patient or their relative for publication of study".

## Competing interests

The authors declare that they have no competing interests.

## Authors' contributions

WA was in charge of the overall care of the patient, reviewed literature, and drafted the manuscript and revised it critically for important intellectual content. BE has participated in the histological diagnosis of the two cases. ASK carried out the literature review. IL participated in the literature review. HE carried out the conception of the case, revised it critically for important intellectual content. All authors read and approved the final manuscript.

## Pre-publication history

The pre-publication history for this paper can be accessed here:

http://www.biomedcentral.com/1471-2342/10/13/prepub
